# The utility of splenic imaging parameters in cardiac magnetic resonance for the diagnosis of immunoglobulin light-chain amyloidosis

**DOI:** 10.1186/s13244-022-01194-8

**Published:** 2022-03-26

**Authors:** Niki Lama, Alexandros Briasoulis, Efstratios Karavasilis, Kimon Stamatelopoulos, Angeliki Chasouraki, Efthymia Alexopoulou, Stavros Spiliopoulos, Foteini Theodorakakou, Meletios Athanasios Dimopoulos, Efstathios Kastritis, Nikolaos L. Kelekis

**Affiliations:** 1 Research Unit of Radiology and Medical Imaging, 2nd Department of Radiology, Medical School, Faculty of Medicine, National and Kapodistrian University of Athens, Papadiamantopoulou 19, 11528 Athens, Greece; 2 Department of Clinical Therapeutics, Medical School, Faculty of Medicine, National Kapodistrian University of Athens, Vasilissis Sofias 80, 11528 Athens, Greece

**Keywords:** Amyloidosis, CMR, Spleen involvement

## Abstract

**Objectives:**

Cardiac magnetic resonance (CMR) imaging is a key test in the diagnosis of cardiac amyloidosis (CA). Extracardiac involvement is common in light chain (AL) amyloidosis and MRI findings may assist in its diagnosis. We sought to investigate the utility of splenic CMR parameters for the diagnosis of CA.

**Methods:**

Thirty-four patients with AL amyloidosis and 32 patients with severe left ventricular hypertrophy in the setting of aortic stenosis (LVH-AS) who completed 3T cardiac MRI at the time of their diagnosis of AL or LVH-AS were assessed with T1, T2 (modified Look-Locker inversion recovery), extracellular volume (ECV) mapping, and late gadolinium enhancement (LGE) imaging of the heart and spleen.

**Results:**

Age, left ventricular mass index, wall thickness, ejection fraction, and splenic dimensions did not differ significantly between groups. All AL patients had cardiac involvement. T1 and T2 spleen mapping did not differ significantly between groups but AL patients had higher median ECV in the spleen than in LVH-AS (AL 46.9%, LVH-AS: 31%, *p* < 0.001), and significantly lower short tau inversion recovery ratio (AL: 1.7, LVH-AS: 2.7, *p* < 0.001) both with very good diagnostic performance to diagnose AL. We identified 16 AL patients with spleen involvement and 16 without. Spleen ECV and “normalized” spleen ratio, defined as the ratio of spleen LGE to muscle values exhibited strong correlation and had excellent diagnostic performance to discriminate those with spleen involvement.

**Conclusion:**

Our findings show that spleen CMR parameters can identify spleen involvement in AL patients and differentiate them from those without AL amyloidosis.

## Keypoints

- Cardiac magnetic resonance (CMR) provides imaging biomarkers to diagnose patients with amyloidosis.
- CMR non-invasive imaging features and parameters can identify spleen involvement in AL patients.
- Extracellular volume (ECV) may demonstrate splenic involvement in AL Amyloidosis.
- Splenic LGE may provide an alternative and/or additional parameter to ECV.

## Background

Systemic amyloidosis represents a complex and heterogeneous group of diseases, defined by the deposition of abnormal proteins, in different tissues and organs, with main consequence organs failure. Classification of amyloidosis is based on the amyloid protein type, and up to date 36 different have been identified [1]. The deposition is mainly extracellular and is recognizable by Congo red stain under polarized light despite the implicated type of protein [1]. Light chain (AL), reactive (AA), mutant or wild type transthyretin (ATTR), fibrinogen (AFib) and apolipoprotein A-I (ApoA1) are the most usual types of systemic amyloidosis [2], with AL amyloidosis being the most frequently diagnosed in developed world [1]. Target organ damage may differ, in each type of amyloidosis. Establishment of amyloid type is crucial as it has direct implications in treatment choice and prognosis. Commonly, the most sensitive biopsy site is the one of affected organs, but less invasive biopsy sites such as the abdominal fat and bone marrow are performed first [2]. Although the diagnosis and typing of amyloidosis requires histological confirmation, imaging modalities such as magnetic resonance imaging (MRI) are emerging as non-invasive methods of diagnosis and typing of amyloidosis [3]. Splenic amyloid deposition appears only in a percentage of patients with certain types of amyloidosis, most commonly in AL amyloidosis and AA amyloidosis, Amyloid fibrils may infiltrate the splenic cords or the splenic blood vessels. Until recently splenic involvement in amyloidosis was underestimated due to the absence of specific clinical manifestations and limited availability of imaging centers that perform 123I SAP scintigraphy, which is a highly sensitive imaging technique to detect the distribution of amyloid deposition in human body organs [4, 5]. Spleen biopsy is the “gold standard” diagnostic method but also an invasive technique with high risk of complications [6].

Therefore, there is an up growing demand for appropriate imaging techniques able to identify involved organs, including spleen. MRI parameters such as diffuse low signal intensity of spleen on T2 weighted images and the increased spleen extracellular volume suggest splenic involvement [4, 7]. The aim of our study is to present features of splenic involvement in patients with cardiac AL amyloidosis (the amyloidosis type with high rates of splenic involvement), identified by cardiac magnetic resonance (CMR) and highlight their role in diagnosis and type of amyloidosis. For this purpose, we compared a newly diagnosed cardiac AL amyloidosis cohort with patients who exhibited severe left ventricular hypertrophy in the setting of aortic stenosis.

## Methods

### Participants

This is a retrospective, single-center analysis approved by the Institutional Review Board that included 34 consecutive patients with cardiac AL amyloidosis. All patients were treated at Department of Clinical Therapeutics, at Alexandra General Hospital of National Kapodistrian University of Athens between September 2020 through November 2021. This is a national high-volume reference center of amyloidosis. The diagnosis of cardiac amyloidosis was based on a combination of typical features on echocardiography, 99 m technetium phyrophosphate scintigraphy, cardiac magnetic resonance imaging and histologically proven systemic AL amyloidosis according to current international recommendation [8]. Patients were diagnosed, treated, and followed prospectively in our center by a group of Oncologists and Cardiologists specialized in CA. Thirty-two age-matched patients with severe left ventricular hypertrophy in the setting of aortic stenosis (LVH-AS) were also recruited as a control group. None of the selected patients with severe AS (aortic stenosis), presented any of the clinical red flags or fulfilled CMR criteria for cardiac amyloidosis [9–11]. The study was approved by the Local Ethical Committee and was conducted in accordance with the Declaration of Helsinki. All participants provided informed consent to participate.

### MRI acquisition protocol

All participants underwent a CMR examination at Research Unit of Radiology of 2nd Department of Radiology of National and Kapodistrian University of Athens, using a 3.0T MRI Philips (Achieva TX) manufactured scanner equipped with a 16-channel XL-Torso2 anterior and posterior receiver coils. The imaging protocol included cine imaging using a retrospectively ECG gated balanced turbo field echo (bTFE) to assess left ventricular (LV) and right ventricular (RV) function, mass and dimensions (TE/TR = 1.5/3 ms, slice thickness = 8 mm without gap, flip angle = 400, acquisition matrix = 185 × 183); a ECG triggered black blood T2 short tau inversion recovery (STIR) to assess myocardium edema (TE/TR = 75 ms/2 RR intervals, slice thickness = 10 mm, gap = 1 mm, flip angle = 900, inversion time delay = 210 ms, acquisition matrix = 200 × 141); a three slices ECG triggered mutli echo turbo spin echo combined with black blood pre-pulse to create T2 map images (TR = 2 RR intervals, number of echoes = 8, ΔTE = 7.7 ms, acquisition matrix = 152 × 148); a ECG triggered phase sensitive inversion (PSIR) (TE/TR = 3.0/6.1 ms, acquisition matrix = 200 × 148, slice thickness = 10 mm with gap = 2 mm, IR value selected after Look-Locker acquisition) 5 and 10 min after the gadolinium injection to assess the myocardium viability; a 2D retrospectively ECG gated phase contrast sequence to quantify the flow of aorta and pulmonary vessel (TE/TR = 4.8/2.8 ms, flip angle = 100, slice thickness = 8 mm). Three slices native and late gadolinium enhanced (LGE) T1 maps were also acquired using modified Look-Locker Inversion recovery (MOLLI) sequence to estimate structural lesions and calculate ECV map considering the hematocrit levels (3slices, TE/TR = 1.03/2.2, slice thickness = 10 mm with gap = 10 mm, acquisition matrix = 120 × 180).

### CMR analysis

**Fig. 1:**
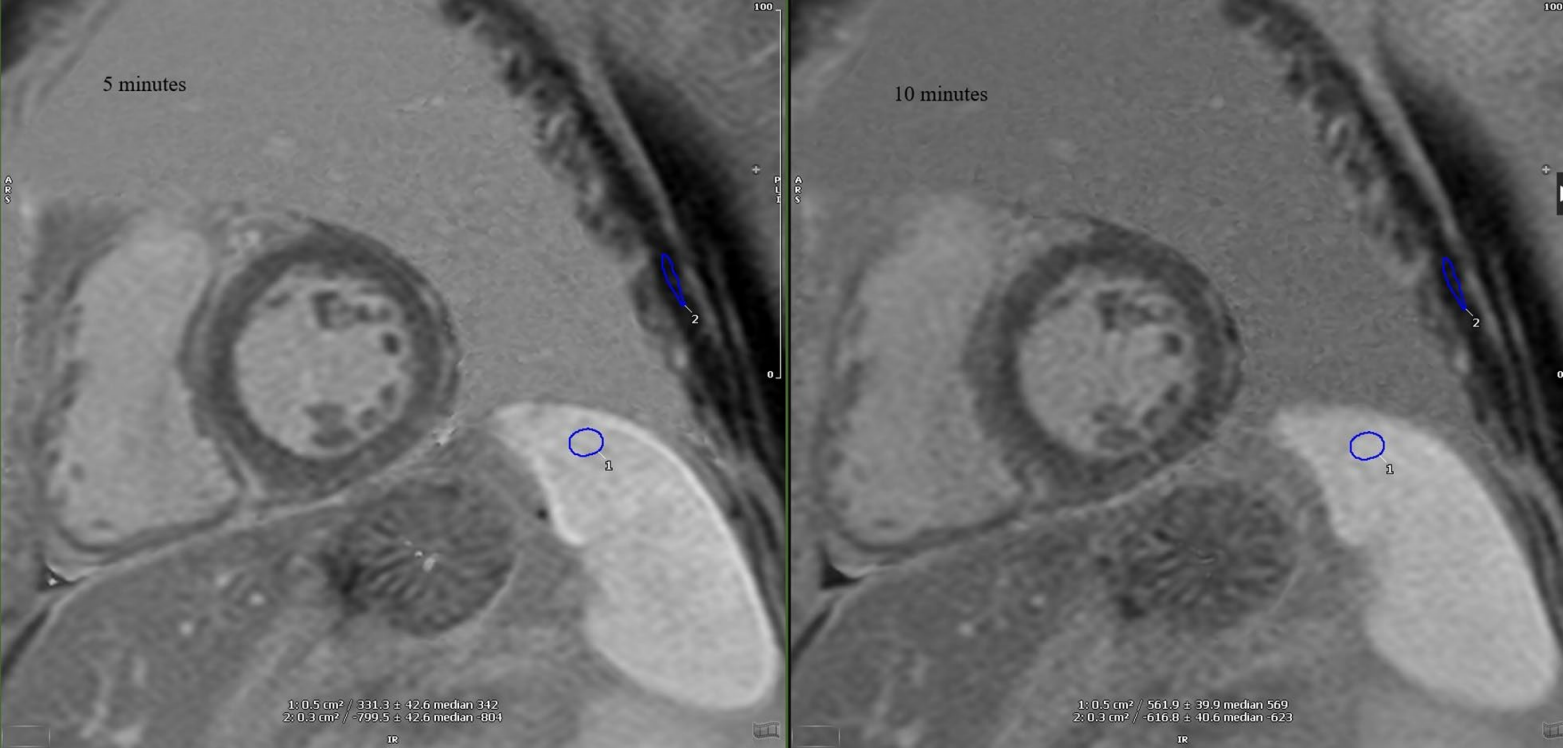
Short-axis PSIR (phase sensitive inversion recovery) images at 5- and 10-min post contrast enhancement in patient with AL Amyloidosis. Regions of interest were drawn in the Spleen and adjacent Serratus anterior muscle, on both images, avoiding vessels, lesions, and artifacts. The “normalized” spleen ratio was defined as the calculated ratio of spleen values to the adjacent muscle values

CMR analysis was performed by an experienced to CMR imaging radiologist and one MRI physicist blinded to participants’ clinical history using the commercially available software (Circle cmr42 release 5.11.4; Circle Cardiovascular Imaging, Calgary, Canada). Left ventricular endo- and epicardial borders were manually outlined in the short-axis slices at the end-diastolic and end-systolic phases. Left ventricular features and function, including LV wall thickness, wall mass, ejection fraction, end-systolic volume, end-diastolic volume and stroke volume, were computed based on short-axis slices. Native and post contrast T1 myocardial relaxation images were firstly manually segmented drawing endocardial and epicardial contours and then were co-registered to eliminate motion-related artifacts, using CVI42 software. Subsequently, the automatically derived global T1, extracellular volume (ECV) and R2 maps were visually checked for the presence of artifacts. Regions of interest were drawn in artifact free spleen area on the short-axis T1, T2 and ECV maps. Regions of interest were also drawn by two experienced raters in spleen and adjacent muscles (preferable Serratus anterior muscle) on short-axis PSIR (phase sensitive inversion recovery) images at 5- and 10-min post contrast enhancement, avoiding vessels, lesions, and artifacts (Fig. 1) and the “normalized” spleen ratio, defined as the ratio of spleen LGE to adjacent muscle values was calculated [12]. Intensity normalization using the signal value of the adjacent muscle was performed to eliminate the signal discrepancy between different subjects, MR scanners or sequences’ configuration. The same methodology was applied on T2-weigted STIR images to calculate the normalized STIR spleen ratio. Interobserver agreement was estimated with intraclass correlation coefficient (ICC). Raters were worked independently and were blinded to the measurements of each other.

### Spleen involvement

The diagnosis of splenic involvement on CMR was based on the findings and criteria introduced by Chacko et al. [4]. The authors showed that extracellular volume (ECV) measurements obtained during routine CMR in patients with suspected amyloidosis can identify and measure the magnitude of amyloid infiltration in the spleen. The findings were validated with the gold standard method which was serum amyloid P component. In their work a cutoff ECV of 38.5% was proposed. In our cohort, we used spleen ECV as well as spleen LGE to exclude spleen involvement. None of the LVH-AS patients exhibited any evidence of spleen involvement. Therefore, we used the highest ECV value of the LVH-AS cohort which was 40%, as a cutoff to exclude or confirm spleen involvement in AL patients. This cutoff was confirmed in a sample of ten healthy volunteers who underwent CMR and served as controls. All exhibited ECV values less than 40%.

### Statistical analysis

Shapiro-Francis test was applied to determine if the estimated parameters are well-modeled by a normal distribution. Continuous variables that are significantly skewed from normal distribution are summarized using median and inter-quartile range. Differences between groups are tested using t-tests and chi-square tests for continuous variables and categorical variables, respectively. Skewed data are tested with the Kruskal–Wallis test. Spearman’s correlation coefficients were calculated to measure the strength and direction of association that exist among CMR parameters. We evaluated the diagnostic utility of spleen CMR parameters for the diagnosis of AL amyloidosis relative to LVH-AS. Optimal cut off for these parameters were derived as the value that minimized the square of the difference between sensitivity and specificity. The diagnostic performance of these parameters was then assessed using probability statistics and ROC analysis. All analyses were conducted using STATA 17.0 with 2-tailed level of significance set at 0.05.

## Results

### Patient characteristics

We studied 66 patients (34 with AL amyloidosis with cardiac involvement and 32 with LVH-AS) who underwent CMR at the time of diagnosis of amyloidosis or LVH-AS. Age (AL: 67.5 years vs. LVH-AS 68 years, *p* = 0.98), body surface area (1.9 m^2^ for both groups) and gender (AL 61.9% vs. LVH-AS 66.7%, *p* = 0.2) did not differ significantly between groups. Systolic blood pressure but not diastolic blood pressure was significantly higher among LVH-AS patients than AL (systolic: *p* = 0.009, diastolic *p* = 0.14).

### CMR parameters

**Fig. 2:**
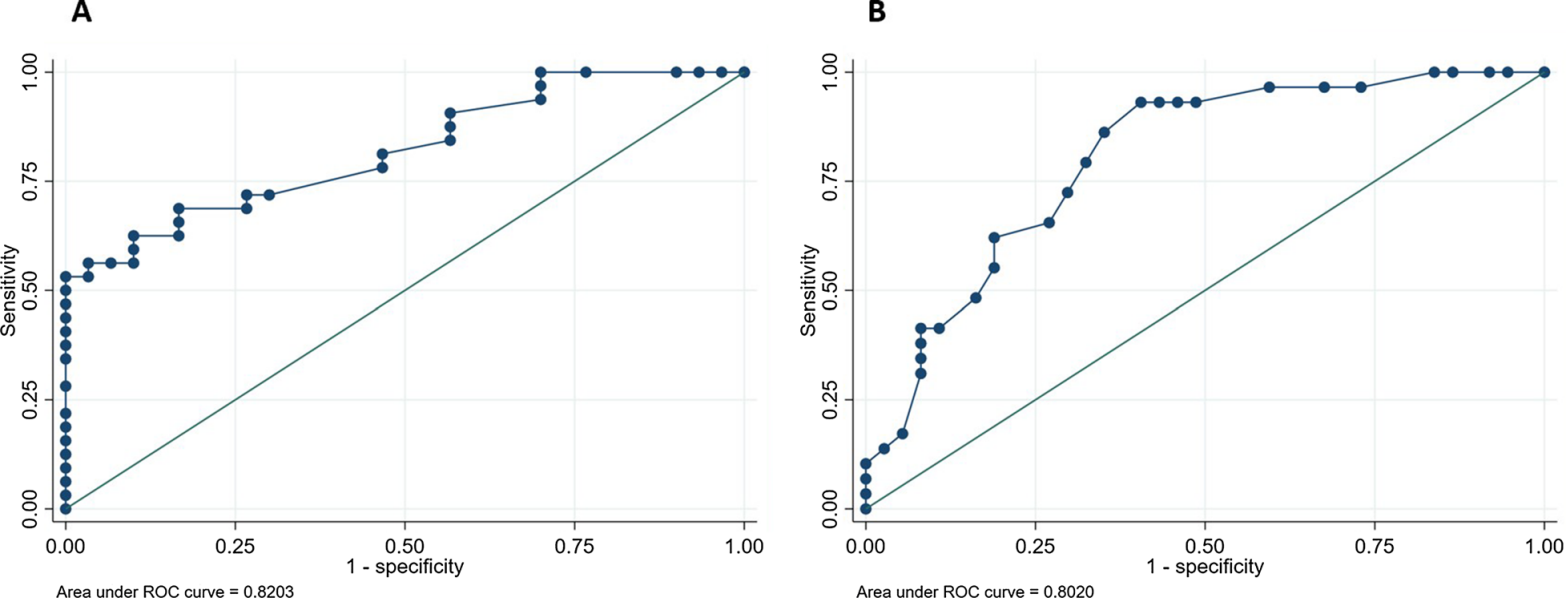
**A** Receiver operating characteristic curve (ROC) of the spleen ECV % between those with AL amyloidosis and left ventricular hypertrophy due to aortic stenosis, **B** ROC curve of spleen STIR ratio between those with AL amyloidosis and left ventricular hypertrophy due to aortic stenosis

Baseline characteristics of cardiac structure and function are presented in Table 1. Patients with AL amyloidosis had similar left ventricular wall thickness, mass index, cardiac index and ejection fraction compared with LVH-AS patients, but significantly higher left ventricular dimensions. Unlike LVH-AS, all AL patients had typical late gadolinium enhancement T1 and T2 spleen mapping did not differ significantly between AL and LVHAS patients (T1 mapping in AL 1392.5 ms vs. LVH-AS 1391 ms, *p* = 0.9, T2 mapping in AL 80 ms vs. LVH-AS 79.5 ms, *p* = 0.8). However, we found significantly higher ECV in the spleen of AL patients than in LVH-AS (median ECV 46.9%, interquartile range [IQR] 32–60 in AL vs. 31%, IQR 29–34 in LVH-AS, *p* < 0.001), and significantly lower short tau inversion recovery (STIR) ratio in AL (STIR: 1.7 IQR 1.4–2.5 in AL vs. 2.7 IQR 2.3–3.5 in LVH-AS, *p* < 0.001). Spleen ECV had very good diagnostic performance to discriminate between AL and LVH-AS with area under the curve AUC = 0.82, 95% Confidence Intervals 0.72–0.92 (Fig. 2). A cut-off point of 34.15% had sensitivity of 69% and specificity of 83% to diagnose AL. Also, spleen STIR ratio exhibited very good diagnostic performance to discriminate between AL and LVH-AS with area under the curve AUC = 0.8, 95% Confidence Intervals 0.7–0.9. A cut-off point of 2.15% had sensitivity of 86% and specificity of 65% to diagnose AL.

**Table 1:** Participants’ demographic, clinical and MRI parameters and their statistical analysis

Variables	Cardiac amyloidosis (*n *= 34)	LVH in AS (*n* = 32)	*p* value between CA and LVH-AS
Age (IQR)	67.5 (60–73)	68 (59–76)	0.98
Body surface area (IQR)	1.9 (1.6–2)	1.9 (1.8–2)	0.28
SBP, mmHg (IQR)	116 (110–130)	130 (120–145)	0.009
DBP, mmHg (IQR)	70 (70–80)	80 (70–88)	0.14
Males (%)	61.9%	66.7%	0.2
Maximum LV wall thickness, mm (IQR)	15 (12–18)	16 (13–17)	0.9
LV mass/BSA, g/m^2^ (IQR)	77.5 (59–94)	78.3 (59.3–97.8)	0.8
LV EDV, ml (IQR)	144.5 (107–170)	75.4 (57.3–100.1)	< 0.001
LV ESV, ml (IQR)	56.5 (31–74)	29 (14.8–43.4)	0.001
LV SV, ml (IQR)	83 (67–95)	46.6 (39.8–57.3)	0.001
LV CO/BSA, ml/m^2^ (IQR)	3.2 (0.7)	3.2 (1.3)	0.6
LVEF, %	61 (55–71)	65 (54–78)	0.4
T1 spleen, ms (IQR)	1392.5 (1346–1455)	1391 (1325–1445)	0.9
T2 mapping Spleen, ms (IQR)	80 (59–92)	79.5 (71.4–91.5)	0.8
ECV spleen %, (IQR)	47 (32–60)	31 (29–34)	< 0.001
STIR ratio spleen, (IQR)	1.7 (1.4–2.5)	2.7 (2.3–3.5)	< 0.001

*CA* cardiac amyloidosis, *LVH-AS* left ventricular hypertrophy in the setting of aortic stenosis, *BSA* body surface area, *LV* left ventricular, *IQR* interquartile range, *SBP* systolic blood pressure, *DBP* diastolic blood pressure, *EDV* end diastolic volume, *ESV* end systolic volume, *SV* stroke volume, *CO* cardiac output, *EF* ejection fraction, *ECV* extracellular volume, *STIR* short tau inversion recovery

### Spleen involvement

**Fig. 3:**
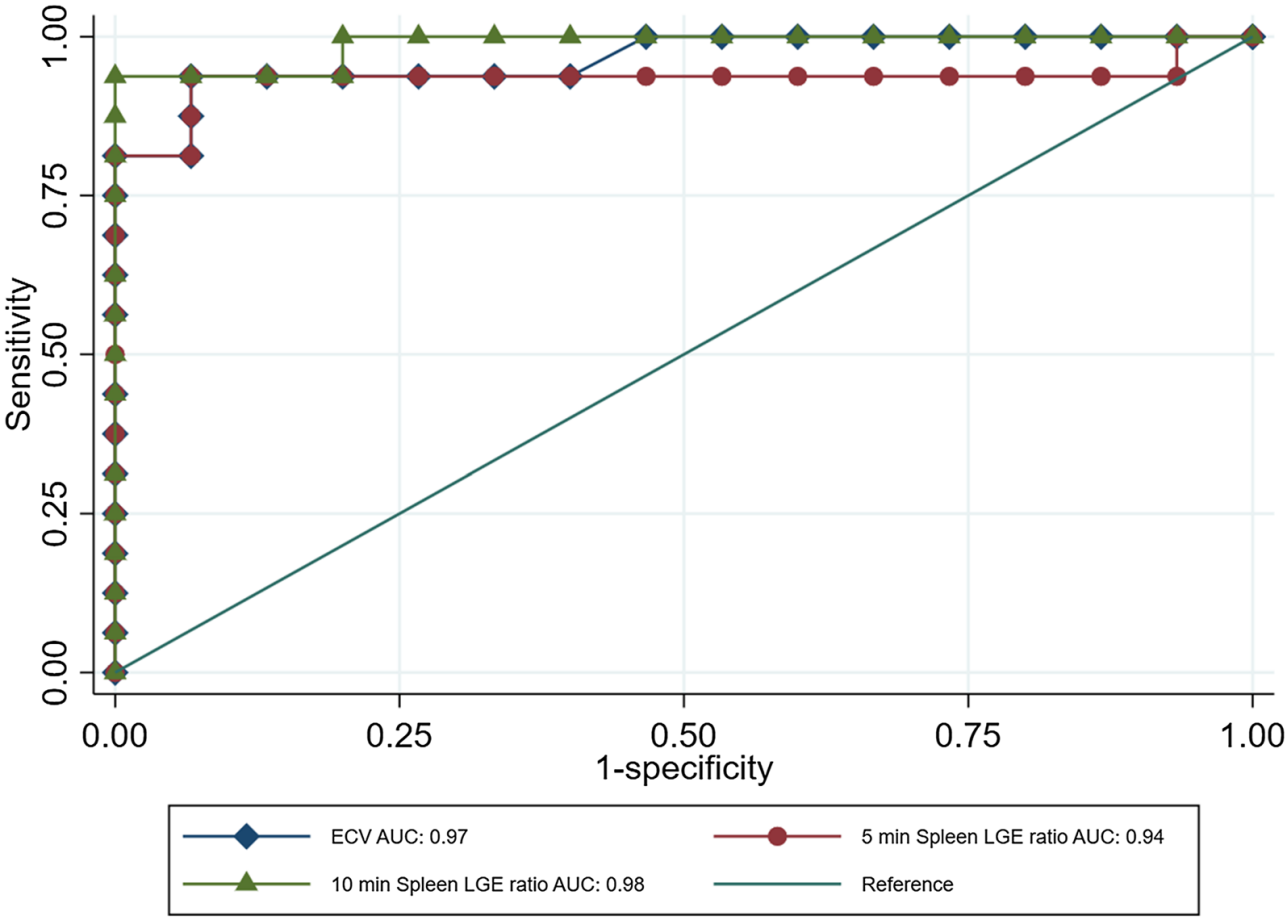
Spleen normalized LGE ratio at 5 and 10 min, and spleen ECV between those AL patients with and without spleen involvement

Among AL amyloidosis patients, we identified 16 patients with spleen involvement and 16 patients without involvement. No patient with LVH-AS had abnormal spleen findings. Spleen ECV was significantly higher in the spleen involvement group (median ECV 60%, IQR 55.5–72.8 vs. 32%, IQR 30.6–36.3, *p* < 0.001). Spleen ECV had excellent diagnostic performance to discriminate spleen involvement with AUC = 0.97, 95% Confidence Intervals 0.91–1 (Fig. 3). Notably, spleen ECV did not differ significantly between LVH-AS and those with AL but no spleen involvement (*p* = 0.78). A cut-off point of 46.9% had sensitivity and specificity of 94%. The normalized spleen LGE ratio at 5 min was significantly lower among those with spleen involvement compared with patients exhibiting no spleen involvement (median spleen involvement − 0.3, IQR − 0.6 to − 0.17 vs. 0.42, IQR 0.29-performance to discriminate spleen involvement with AUC 0.94, 95% Confidence Intervals 0.83–1 (Fig. 3), and no significant difference compared with the AUC of spleen ECV (*p* = 0.87). A cut-off point of 0.01 had sensitivity and specificity of 94% to diagnose spleen involvement.

Similarly, the normalized spleen LGE ratio at 10 min was significantly lower among those with spleen involvement compared with patients exhibiting no spleen involvement (median spleen involvement − 0.45, IQR − 1.12 to − 0.07 vs. 0.47, IQR 0.43–0.59, *p* < 0.001). Normalized spleen LGE ratio at 10 min had excellent diagnostic performance to discriminate spleen involvement with AUC 0.98, 95% Confidence Intervals 0.95–1, and no significant difference compared with the AUC of spleen ECV (*p* = 0.37). A cut-off point of 0.35 had sensitivity of 88% and specificity of 100% to diagnose spleen involvement.

There was a significant negative correlation between spleen ECV and LGE spleen ratio at 5 min in AL patients (Spearman’s rho = − 0.7, *p* < 0.001). Also, we found significant correlation between ECV and LGE spleen ratio at 10 min in AL patients (Spearman’s rho = − 0.72, *p* < 0.001). We found no differences in myocardial native T1 and ECV between those with and without spleen involvement (*p* = 0.35 and 0.1, respectively). The inter-rater agreement was substantial for all metrics (0.9, *p* < 0.001).

## Discussion

**Fig. 4:**
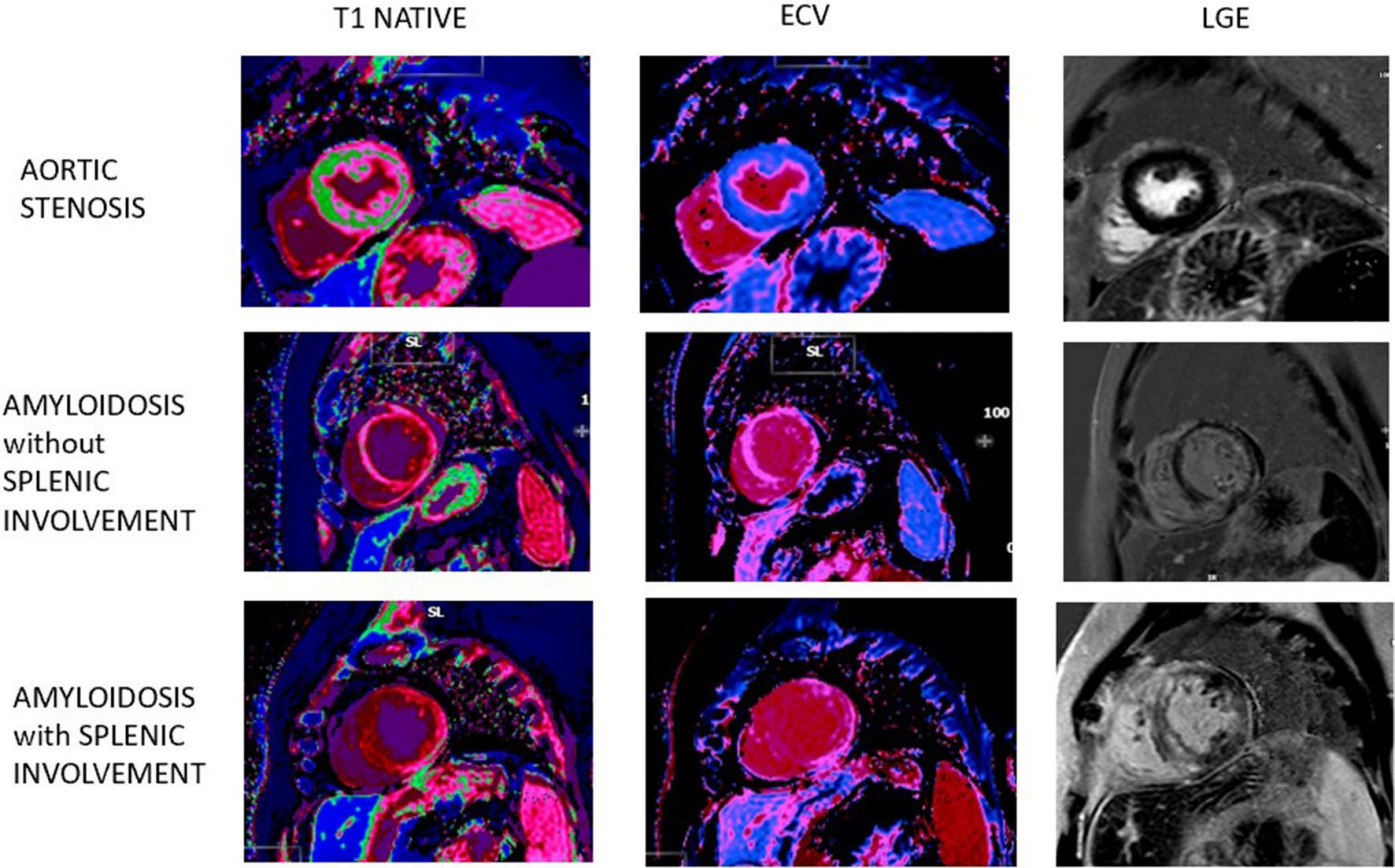
Table presenting the differences in myocardial and splenic values in a patient with Aortic Stenosis, and patients with Cardiac Amyloidosis with and without splenic involvement, in T1 NATIVE map and ECV map, in correspondence to late gadolinium enhancement (LGE) images

The salient findings of this analysis of a single center contemporary cohort of AL amyloidosis patients evaluated with CMR at the time of diagnosis can be summarized as follows: (1) imaging of the spleen and measurement of several parameters including T1, T2 mapping and ECV was feasible in all AL and LVH-AS patients, (2) no differences in T1 and T2 mapping were identified between groups, (3) ECV and STIR ratio of the spleen was significantly higher in AL patients with very good diagnostic performance to discriminate between AL and LVH-AS (Fig. 2), (4) normalized spleen LGE ratio at 5 and 10 min, easily measurable markers, were significantly lower among those with spleen involvement and had excellent diagnostic performance to detect spleen involvement (Fig. 3).

AL amyloidosis is the most frequent systemic amyloidosis in the developed countries [2]. In SAP scintigraphy splenic amyloid was detected very frequently (80%) in AL amyloidosis, but seldom in ATTR amyloidosis [13]. SAP and MRI are the two most commonly employed methods to reliably assess amyloid involvement of various organs including the heart, liver and spleen [4, 5]. However, limited data are available on imaging patterns and parameters of MRI assessment in cases of spleen involvement by amyloid [4, 7, 14–16]. Unlike other conditions involving the spleen, in AL amyloidosis a more diffuse pattern is expected. A core biopsy of splenic tissue would be associated with prohibitive bleeding risk; hence, the utilization of advanced imaging techniques may facilitate the identification of splenic involvement and the diagnosis of AL and exclude the diagnosis of LVH from other causes (including ATTR) unlikely. Our main hypothesis was to examine whether extracardiac imaging manifestations of AL amyloidosis can be used to differentiate this condition from other causes of LVH. Therefore, we focused on the spleen as a target organ. Imaging of this can be routinely performed during a CMR protocol and diffuse involvement of the spleen is expected in AL based on the systemic deposition of protein derived from immunoglobulin light chain fragments. We confirmed the feasibility of routine use of ECV as well as LGE to detect changes in splenic tissue. Although native T1 splenic measurements were comparable between groups, ECV% and STIR ratio proved to be excellent differentiation tools between the two conditions studied here.

Moreover, we noticed a visual correlation between ECV measurements and splenic enhancement during early and late gadolinium phase (Fig. 4). Spleen involvement was characterized by a diffuse hyperenhancement in post contrast images. The use of LGE spleen ratio at 5 and 10 min, widely available and easy to measure markers, confirmed our hypothesis and previous literature that spleen parameters may accurately identify spleen involvement [4] and facilitate diagnosis of AL amyloidosis as patients with ATTR amyloidosis or no amyloidosis but severe LVH exhibit no spleen involvement.

Recently, an analysis of 533 patients [4] with suspected systemic amyloidosis who underwent SAP scintigraphy and CMR with T1 mapping showed that among 363 confirmed patients with systemic amyloidosis, 148 patients had liver and/or spleen involvement and among those almost all had AL and the majority had cardiac involvement. This study of a large cohort of patients with systemic amyloidosis demonstrated for the first time that liver and spleen ECV mapping obtained during CMR can identify the presence and measure the magnitude of amyloid infiltration in the liver and spleen. This additional information, available readily, has potential not only to highlight the type of amyloid, since splenic amyloid is frequent in AL but does not occur in the prevalent wild-type transthyretin amyloidosis, but also to determine prognosis and measure response to treatment. Our findings build upon the recently published data as we confirm the utility of spleen ECV and add a novel, easy to measure marker to the CMR parameters used in the evaluation of these patients.

This study is subject to the limitations inherent to any single-center study with observational, retrospective design and nonrandomized treatment assignment. First, the sample size of this study is relatively small. However, it should be taken into consideration that AL is a rare condition. Moreover, external validation of our findings is necessary before implementation into clinical practice. Overall, the findings should be viewed as hypothesis generating and further multicenter analyses of larger sample sizes are warranted, to incorporate cardiac and extracardiac CMR parameters into the routine criteria for the diagnosis of CA. The main strength of our study is the detailed analysis of CMR parameters in a well-defined cohort of AL CA patients treated in a referral center for AL by a group of physicians specialized in this field.

## Conclusion

In conclusion, we identified CMR biomarkers based on spleen measurements with very good diagnostic performance to differentiate between AL and LVH-AS and novel simple spleen metrics based on LGE of spleen with excellent diagnostic performance to detect spleen involvement in AL amyloidosis. Spleen involvement and respective CMR findings could be incorporated in the diagnostic process and subtyping of amyloidosis. A comprehensive validation of cardiac and extracardiac (splenic) CMR parameters for diagnostic and prognostic purposes is warranted.

## Data Availability

The datasets used and analyzed during the current study are available from the corresponding author on reasonable request.

## Abbreviations

AA: (Secondary) serum protein A amyloidosis
AFib: Fibrinogen amyloidosis
AL: Amyloidosis light chain
ApoA1: Apolipoprotein A1
AS: Aortic stenosis
ATTR: Wild type transthyretin amyloidosis
BSA: Body surface area
bTFE: Balanced turbo field echo
CA: Cardiac amyloidosis
CMR: Cardiac magnetic resonance
CO: Cardiac output
DBP: Diastolic blood pressure
ECV: Extracellular volume
EDV: End diastolic volume
EF: Ejection fraction
ESV: End systolic volume
ICC: Intraclass correlation coefficient
IQR: Interquartile range
LGE: Late gadolinium enhancement
LV: Left ventricle
LVH-AS: Left ventricular hypertrophy in the setting of aortic stenosis
MOLLI: Modified Look-Locker Inversion recovery
MRI: Magnetic resonance imaging
PSIR: Phase sensitive inversion recovery
ROI: Region of interest,
RV: Right ventricle
SBP: Systolic blood pressure
STIR: Short tau inversion recovery
SV: Stroke volume
